# Analytical Variables Affecting Analysis of F_2_-Isoprostanes and F_4_-Neuroprostanes in Human Cerebrospinal Fluid by Gas Chromatography/Mass Spectrometry

**DOI:** 10.1155/2013/810915

**Published:** 2013-07-10

**Authors:** Hsiu-Chuan Yen, Hsing-Ju Wei, Ting-Wei Chen

**Affiliations:** Department of Medical Biotechnology and Laboratory Science, College of Medicine, Chang Gung University, Tao-Yuan 333, Taiwan

## Abstract

F_2_-isoprostanes (F_2_-IsoPs) are a gold marker of lipid peroxidation *in vivo*, whereas F_4_-neuroprostanes (F_4_-NPs) measured in cerebrospinal fluid (CSF) or brain tissue selectively indicate neuronal oxidative damage. Gas chromatography/negative-ion chemical-ionization mass spectrometry (GC/NICI-MS) is the most sensitive and robust method for quantifying these compounds, which is essential for CSF samples because abundance of these compounds in CSF is very low. The present study revealed potential interferences on the analysis of F_2_-IsoPs and F_4_-NPs in CSF by GC/NICI-MS due to the use of improper analytical methods that have been employed in the literature. First, simultaneous quantification of F_2_-IsoPs and F_4_-NPs in CSF samples processed for F_4_-NPs analysis could cause poor chromatographic separation and falsely higher F_2_-IsoPs values for CSF samples with high levels of F_2_-IsoPs and F_4_-NPs. Second, retention of unknown substances in GC columns from CSF samples during F_4_-NPs analysis and from plasma samples during F_2_-IsoPs analysis might interfere with F_4_-NPs analysis of subsequent runs, which could be solved by holding columns at a high temperature for a period of time after data acquisition. Therefore, these special issues should be taken into consideration when performing analysis of F_2_-IsoPs and F_4_-NPs in CSF to avoid misleading results.

## 1. Introduction

 Reliable assessment of oxidative stress *in vivo* has been important for investigating the roles of oxidative stress in the pathogenesis or progression of diseases [1]. F_2_-isoprostanes (F_2_-IsoPs) are prostaglandin (PG)-like compounds derived from lipid peroxidation of arachidonic acid (AA, C20:4 *ω*-6), which is abundant in all kinds of cells, initiated by free radicals independent of the cyclooxygenase pathway. They are initially formed as esterified form on phospholipids and can be released into surrounding body fluids to become free form via the action of enzymes with the phospholipase-like activities [2–5]. There are four regioisomers of F_2_-IsoPs, but the 5-series and 15-series regioisomers are the major regioisomers formed *in vivo* [6]. F_2_-IsoPs have been well recognized as the most reliable and specific marker of lipid peroxidation *in vivo* and is a widely used marker of oxidative damage due to several favorable characteristics [7]. On the other hand, F_4_-neuroprostanes (F_4_-NPs) are generated from the lipid peroxidation of docosahexaenoic acid (DHA, C22:6 *ω*-3) via similar mechanisms, but eight regioisomers are produced [8]. As shown by Yin et al., 4-series and 20-series regioisomers are the most abundant regioisomers of F_4_-NPs generated *in vitro* and *in vivo* [9]. Measurement of F_4_-NPs in CSF or brain tissue has been considered as a more selective marker for neuronal oxidative damage because DHA is enriched in neurons [10]. 

 Gas chromatography/negative-ion chemical-ionization mass spectrometry (GC/NICI-MS) is the most sensitive and robust method for routine quantification of F_2_-IsoPs and F_4_-NPs in biological samples [11, 12], which is required for body fluids with low levels of free F_2_-IsoPs and F_4_-NPs or limited availability, such as cerebrospinal fluid (CSF). Different assay platforms and further modifications for GC/NICI-MS analysis of F_2_-IsoPs and F_4_-NPs were present in the literature as discussed in our previous paper [13], but most of the them were modified from procedures from the groups of Roberts or Morrow [8, 14, 15]. As previously reviewed by us, methods involving liquid chromatography/mass spectrometry (LC/MS) were superior than the GC/NICI-MS method primarily in the aspect of identification of different regioisomers, but it could only be used for quantification of highly abundant free F_2_-IsoPs in urine or total levels of F_2_-IsoPs in plasma, which consisted of abundant esterified F_2_-IsoPs from lipoproteins, due to its lower sensitivity [16]. Moreover, the unique lipid chromatography/atmospheric pressure chemical ionization mass spectrometry (LC/APCI-MS) method developed by Yin et al. was only used to identify different regioisomers of F_4_-NPs in liver tissue and DHA oxidized *in vitro* [9], whereas detection of F_2_-IsoPs and F_4_-NP in CSF in the literature so far, including our studies, was only performed by the GC/NICI-MS method [8, 13, 17–21]. 

 Although the GC/NICI-MS method has been recognized as the reference method to quantify F_2_-IsoPs and F_4_-NP levels in body fluids, several variants of analytical settings exist in the literature for GC/MS analysis. For example, the area on silica recovered from thin-layer chromatography (TLC) plates should be smaller for F_2_-IsoPs analysis than that for F_4_-NPs analysis to obtain better chromatographic separation [13, 17], but Corcoran et al. simultaneously analyze total (free plus esterified) levels of F_2_-IsoPs, F_4_-NPs, and isofurans, another product of lipid peroxidation from AA, in human CSF without showing any chromatogram when they employed a very different method without the step of TLC purification [18]. Because the amount of CSF needed for F_4_-NPs is much higher for F_2_-IsoPs analysis, it is also a tempting idea to perform simultaneous analysis of the same sample when the amount of CSF samples available is very little. On the other hand, during our previous investigation on F_4_-NPs of human CSF, we noticed obvious retention of unknown compounds from the previous samples on GC columns at *m/z* 593.5, which was used to detect F_4_-NPs, by using the temperature ramp commonly indicated to analyze F_2_-IsoPs or F_4_-NPs [15, 22]. We speculated that when this problem was not noticed, the results of F_4_-NPs quantification in CSF might not be reliable. Accordingly, we also wondered whether analysis of F_4_-NPs in CSF could also be affected by the retention effect following analysis of F_2_-IsoPs of CSF or other body fluids since it was very common to analyze different samples for either F_2_-IsoPs or F_4_-NPs interchangeably during routine operation.

 In this report, we first investigated whether simultaneous analysis of F_2_-IsoPs and F_4_-NPs for CSF samples processed for F_4_-NPs analysis is feasible by comparing chromatograms and results of F_2_-IsoPs quantification from CSF samples processed for F_2_-IsoPs and F_4_-NPs separately. Furthermore, we systematically examined whether significant amount of substances was retained in GC columns from previously injected samples by simultaneously recording chromatograms at *m/z* 569.4, *m/z* 593.5, and *m/z* 573.4, which were masses employed to detect F_2_-IsoPs, F_4_-NPs, and [^2^H_4_]-15-F_2*t*_ -IsoP (internal standard), respectively, after analyzing F_2_-IsoPs and F_4_-NPs in CSF or analyzing F_2_-IsoPs in urine and plasma. We also evaluated whether the peaks from the retained substances could overlap with the peaks for quantification of F_2_-IsoPs, F_4_-NPs, or [^2^H_4_]-15-F_2*t*_-IsoP (internal standard) at their corresponding masses. Finally, the beneficial effect of additional holding of the column at a high temperature after regular data acquisition in the method setting on removing retained peaks was investigated.

## 2. Materials and Methods

### 2.1. Test Samples

 For the testing in this study, we used three pooled human CSF samples with different levels of F_2_-IsoPs and F_4_-NPs, which were designated as L-CSF, M-CSF, and H-CSF for low levels, medium levels, and high levels of these compounds, respectively. L-CSF sample was from patients with normal pressure hydrocephalus, whereas M-CSF and H-CSF samples were from patients with aneurysmal subarachnoid hemorrhage (aSAH). These CSF samples were pooled from specimen collected during our previous study on aSAH that have been published [13, 17], in which we showed that CSF samples from aSAH had much higher levels of F_4_-NPs than that from non-aSAH controls including those with normal pressure hydrocephalus [13]. Moreover, one test plasma sample and one test urine sample were also pooled samples from normal subjects used in the previous studies [23].

### 2.2. Sample Processing and GC/MS Detection for Analysis of Free F_2_-IsoPs in CSF, Plasma, and Urine

 The methods of analyzing free F_2_-IsoPs in human body fluids was modified from the procedures described by the Morrow's group [11, 14]. Some of major modifications on solid-phase extraction (SPE) and GC/MS settings have been indicated in our previous publications [13, 17]. In brief, an appropriate volume of body fluids was added into 3 mL of ultrapure water containing the internal standards and the pH was adjusted to pH 3, which was followed by two runs of SPE purification with C_18_ columns and then silica columns. The internal standard used for CSF and plasma was [^2^H_4_]-15-F_2*t*_-IsoP [14], whereas that used for urine was [^2^H_4_]-8-F_2*t*_-IsoP to avoid interferences peaks from endogenous substances in urine samples [24]. [^2^H_4_]-15-F_2*t*_-IsoP and [^2^H_4_]-8-F_2*t*_-IsoP were purchased from Cayman Chemical, which were designated as 8-iso prostaglandin F_2*α*_-d_4_ (catalog number 316350) and iPF_2*α*_-IV-d_4_ (catalog number 316230), respectively, in the catalog of Cayman Chemical. These organic solvents in vials containing the two internal standards purchased from Cayman Chemical were evaporated and resuspended in a fixed amount of ethanol followed by the calibration of true concentration of the internal standard in the ethanol solution by the method of Milne et al. [11]. In this study, 0.6 mL of CSF, 0.5 mL of plasma, and 0.05 mL of urine samples were mixed with 100 pg, 250 pg, and 330 pg of the internal standards, respectively, for each sample preparation. Because the concentrations of M-CSF and H-CSF samples were known to be much higher than L-CSF, only 0.4 mL and 0.24 mL of M-CSF and H-CSF samples, respectively, were used along with 0.6 mL of L-CSF for the test described in this study. 

The elution and washing steps for SPE purification were the same as the method of Milne et al. [14], but 6-mL disposable columns with 500 mg sorbent (J. T. Baker) was used and SPE was operated on a negative-pressure vacuum manifold column processor (J. T. Baker), which was connected to a vacuum pump capable of controlling the speed of SPE flow, to simultaneously process multiple samples [17]. The eluate from C_18_ columns was dried very briefly with 2 g anhydrous sodium sulfate in glass vials before being processed by silica columns. The analyte in final eluate from silica columns was dried, converted to PFB esters by using PFBB and DIPE, purified by thin-layer chromatography (TLC), and extracted by ethyl acetate according to the methods of Milne et al. [14]. The scarping range on TLC plates was 1 cm above and 1 cm below the TLC standard, methyl ester of PGF_2*α*_. After the second derivatization by *N,O*-bis(trimethylsilyl)trifluoroacetamide (BSTFA) and dimethylformamide (DMF), which was stored over CaH_2_, and further dryness by nitrogen gas, the analyte was dissolved in an appropriate amount of undecane, which was also stored on CaH_2_, for further injection into the GC/MS [14]. 

 F_2_-IsoPs in the samples and the internal standards added into the samples were detected at *m/z* 569.4 and 573.4, respectively, by the mode of selected ion monitoring. The ion detected by GC/NICI-MS was the trimethylsilyl ether derivative of the carboxylate anion of F_2_-IsoPs or the internal standard, which has been well illustrated by us [13] or Milne et al. [22]. Amount of F_2_-IsoPs in the samples was quantified by multiplying amount of [^2^H_4_]-15-F_2*t*_-IsoP or [^2^H_4_]-8-F_2*t*_-IsoP added into the samples with the ratio of the peak height of F_2_-IsoPs to that of the corresponding internal standard. Settings and instrumentation of GC/NICI-MS used was the same as what we have previously described [13], in which the 6890 GC/5975 MS and DB-1701 capillary column from Agilent were used, except the inclusion of 2-min holding at 300°C at the end of temperature ramp (190°C to 300°C at 18°C/min) for the acquisition of chromatograms followed by additional holding of the GC column at 280°C with the detector off. The second holding was omitted for some examinations in this study; otherwise, this additional holding time was set as 20 min for routine analysis of CSF and plasma samples and 10 min for urine samples based on the testing results described in this study. 

### 2.3. Sample Processing and GC/MS Detection for Analysis of Free F_4_-NPs in CSF

 The procedures for analysis of free F_4_-NPs in human CSF have been described previously in our publication [13], which were modified from the method of Arneson and Roberts II [15], except that 250 pg of the internal standard [^2^H_4_]-15-F_2*t*_-IsoP was used with 1.5 mL of CSF. For M-CSF and H-CSF, 1 mL and 0.6 mL of samples, respectively, were used and diluted to 1.5 mL with water before adding into 3 mL of water. The whole process and principle are similar to those for analysis of free F_2_-IsoPs in body fluids. One difference was that the washing solvent for silica SPE columns in the second SPE was ethyl acetate [14] and ethyl acetate/heptane (75 : 25) [15] for F_2_-IsoPs analysis and F_4_-NPs, respectively. Another difference was that the scraping area of TLC plates for extraction of F_4_-NPs was 1 cm below and 3 cm above the TLC standard [8, 13] instead of the 2 cm range performed for F_2_-IsoPs analysis described in Section 2.2. Different from other methods described in the literature, we previously established the method by overlaying the chromatograms of CSF samples with that of products from *in vitro* oxidation of DHA at *m/z* 593.5 to identify the range of peaks for quantification of F_4_-NFs [13, 16]. In this study, as for the F_2_-IsoPs analysis, additional 2-min run at 300°C was included for the acquisition of chromatograms. The setting of additional 30-min holding at 280°C without turning the detector on was included in the method for our routine analysis, which was based on the observation from this study, was purposely omitted for some tests in this report. Amount of F_4_-NPs in samples was quantified by multiplying amount of [^2^H_4_]-15-F_2*t*_-IsoP added into samples with the ratio of the peak area of F_4_-NPs at *m/z* 593.5, which was defined by the range of peaks of oxidized DHA, to that of [^2^H_4_]-15-F_2*t*_-IsoP. 

### 2.4. Statistical Analysis

 The significance of the difference for the data between the 2 groups was evaluated using a two-tailed independent *t*-test with SPSS software (SPSS Inc.). The Levene test was also conducted to determine whether *p* values should be obtained under the assumption of equal variance or unequal variance. Statistical significance was considered when *p* values were smaller than 0.05. 

## 3. Results

### 3.1. Results of F_2_-IsoPs Analysis in CSF for Samples Processed for F_2_-IsoPs versus Samples Processed for F_4_-NPs

**Figure 1:**
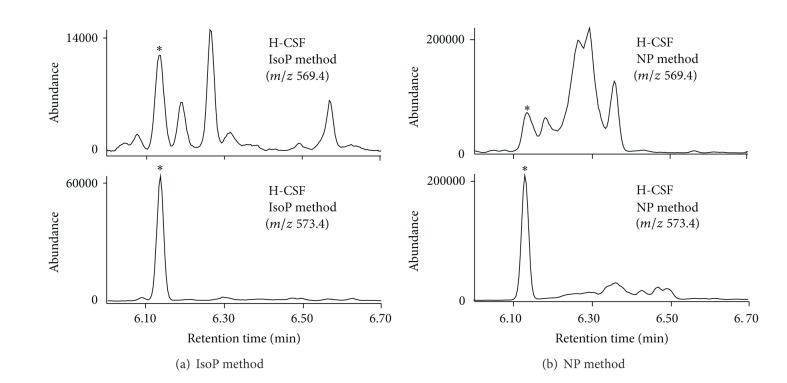
Comparison on the patterns of chromatograms at *m/z* 569.4 for the H-CSF sample processed for F_2_-IsoPs (IsoP method) and for F_4_-NPs (NP method). Because the amount of samples and internal standard used were different for different methods, the abundance of peaks for the internal standard at *m/z* 573.4 was also shown. The peaks of F_2_-IsoPs and [^2^H_4_]-15-F_2*t*_-IsoP at *m/z* 569.4 and *m/z* 573.4, respectively, are indicated by the asterisks.

 To address the question that whether simultaneous analysis of F_2_-IsoPs and F_4_-NPs for the same CSF sample processed for F_4_-NPs analysis was appropriate, we first analyzed F_2_-IsoPs and F_4_-NPs simultaneously for L-CSF, M-CSF, and H-CSF samples processed for F_4_-NPs analysis from three independent replicates of each sample. The results of F_2_-IsoPs quantification were then compared with those for the corresponding three CSF samples processed for F_2_-IsoPs analysis in a separate experiment. As shown by Table 1, we found that differences in mean values of F_2_-IsoPs from samples processed in two different ways relative to the values from samples processed for F_2_-IsoPs were small for L-CSF (5.2%) and M-CSF (1.7%) but were very high for H-CSF (66%). Despite of having the small difference for values of F_2_-IsoPs quantified from two different methods (5.2%), there was statistical significance between values of F_2_-IsoPs for L-CSF processed for F_2_-IsoPs analysis versus for F_4_-NPs analysis. It could be a systematic difference due to amount of CSF samples or internal standards used in two different operations. On the other hand, due to wider TLC scraping ranges for F_4_-NPs analysis, the abundance of the peaks adjacent to the peak of F_2_-IsoPs became much higher for all samples tested. The chromatograms at *m/z* 569.4 of H-CSF processed in two different ways are shown to illustrate such alteration (Figure 1). The results showed that the peak resolution for samples processed for F_4_-NPs was worse due to significant overlap with the adjacent peaks and the baseline was higher for the peak of F_2_-IsoPs compared with that processed for F_2_-IsoPs. 

### 3.2. Evaluation on the Extent of Sample Retention on GC Columns without Additional Holding of the Column at the High Temperature after Analysis of F_4_-NPs for the M-CSF Sample

 For GC/MS settings, all methods that originated from the groups of Morrow and Roberts in the literature for F_2_-IsoPs and F_4_-NPs indicated the temperature ramp of 190 to 290 or 300°C without additional holding [11, 12, 14, 15]. However, during the analysis of F_4_-NPs in human CSF for our previous study on aSAH [13], we noticed that some residual substances on the GC column after each analysis tended to be retained and eluted out together with the next samples, which could be detected at *m/z* 593.5, although the degree of this effect varied from sample to sample. To more systematically examine such effect at different masses, in this study, we first examined chromatograms at *m/z* 569.4, *m/z* 573.4, and *m/z* 593.5 for 4 injections of undecane, indicated as “Wash,” following analysis of F_4_-NPs in CSF. Although *m/z* 569.4 was not used for detection of F_4_-NPs, it was important to monitor all masses used because analysis of F_2_-IsoPs may be performed right after analysis of F_4_-NPs during routine operation.

**Figure 2:**
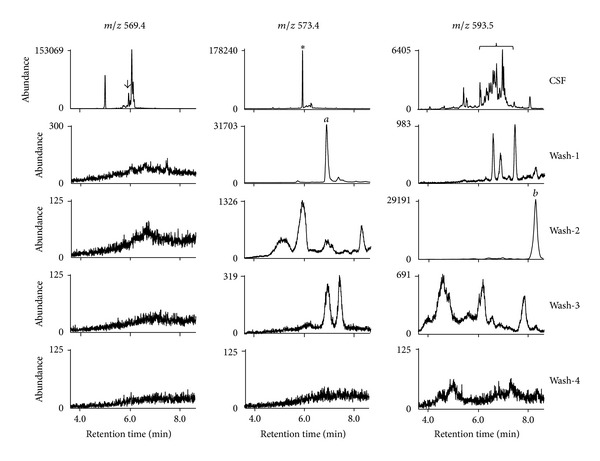
Chromatograms at *m/z* 569.4, *m/z* 573.4, and *m/z* 593.5 for the M-CSF sample processed for F_4_-NPs analysis and the 4 subsequent injections of undecane alone (Wash-1 to Wash-4). The chromatograms in the range of 3.6 min to 8.6 min (the end of data acquisition) were compared. The peak of F_2_-IsoPs is indicated by the arrow. The peak of [^2^H_4_]-15-F_2*t*_-IsoP is indicated by the asterisk. Peak *a* and peak *b* indicated in the text were peaks of the retained substances from the sample with very high abundance. The range of retention time for quantification of F_4_-NPs peaks at *m/z* 593.5, which is defined by that for the peaks of oxidized DHA, is indicated by the brace.

 All the sources and processing of samples were the same for different figures for this test. Only M-CSF was used for this purpose. To simulate the method frequently applied, second holding of the GC column at 280°C with the detector off was not conducted although we included 2-min holding at 300°C at the end of temperature ramp to make the acquisition of chromatograms more complete. As shown by Figure 2, chromatograms in the range of 3.6 min to 8.6 min (the end of data acquisition) of the 4 undecane washes after the injection of M-CSF sample indicated that there were obvious unknown peaks retained in the column from the previous CSF sample at *m/z* 573.4 and *m/z* 593.5 but not *m/z* 569.4. Some peaks of washes were at the retention time for the quantification of target compounds, such as one peak of Wash-2 at *m/z* 573.4 and three peaks of Wash-1 at *m/z* 593.5, although the abundance of the peaks from these washes was much less than that from the CSF sample. Moreover, the peak *a* of Wash-1 at *m/z* 573.4 and the peak *b* of Wash-2 at *m/z* 593.5 were very large although they did not overlap with the retention time of F_4_-NPs peaks. 

**Figure 3:**
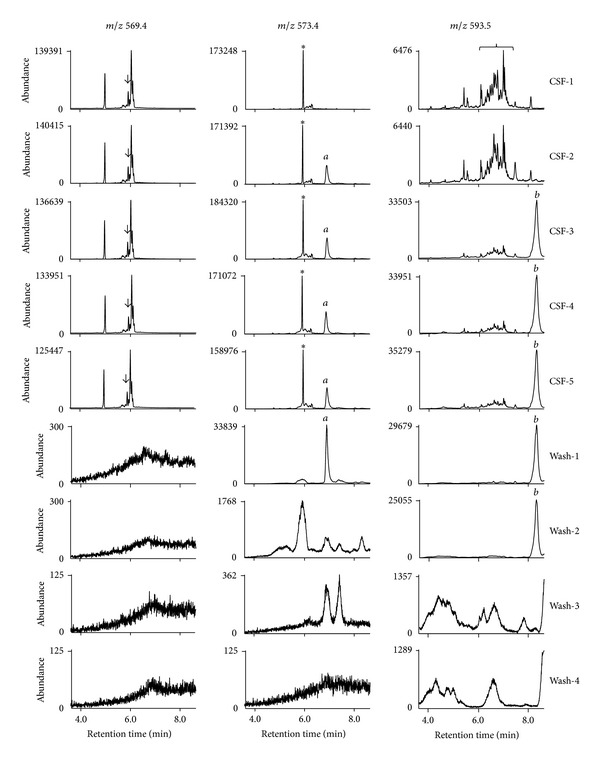
Chromatograms at *m/z* 569.4, *m/z* 573.4, and *m/z* 593.5 for the 5 consecutive injections of the same pooled M-CSF sample processed for F_4_-NPs analysis (CSF-1 to CSF-5) and the 4 subsequent injections of undecane alone (Wash-1 to Wash-4). The chromatograms in the range of 3.6 min to 8.6 min (the end of data acquisition) were compared. The peaks of F_2_-IsoPs at *m/z* 569.4 are indicated by the arrows. The peaks of [^2^H_4_]-15-F_2*t*_-IsoP at *m/z* 573.4 are indicated by the asterisks. The range of retention time for quantification of F_4_-NPs peaks at *m/z* 593.5, which is defined by that for the peaks of oxidized DHA, is indicated by the brace.

 Furthermore, we wondered how the chromatograms would look like and the degree of retention would be when 5 CSF samples were analyzed consecutively without additional holding. To examine this issue, multiple processed M-CSF samples dissolved in undecane were pooled together and multiple injections from the same pooled sample were performed. We found that the large peaks, peak *a* and peak *b*, at *m/z* 573.4 and *m/z* 593.5, respectively, showed up behind the target peaks during the injections of real samples, which were similar to the pattern found in Figure 2 (middle and right panels of Figure 3). Moreover, small but obvious peaks at the retention time for the quantification of target peaks also appeared in Wash-2 at *m/z* 573.4 and 4 Washes at *m/z* 593.5 (middle and right panels of Figure 3). On the contrary, there was no obvious obscure peak beyond background signals in the chromatograms at *m/z* 569.4 (left panel of Figure 3). 

### 3.3. Evaluation on the Extent of Sample Retention on GC Columns without Additional Holding of the Column at the High Temperature after Analysis of F_2_-IsoPs for the M-CSF, Plasma, and Urine Samples

**Figure 4:**
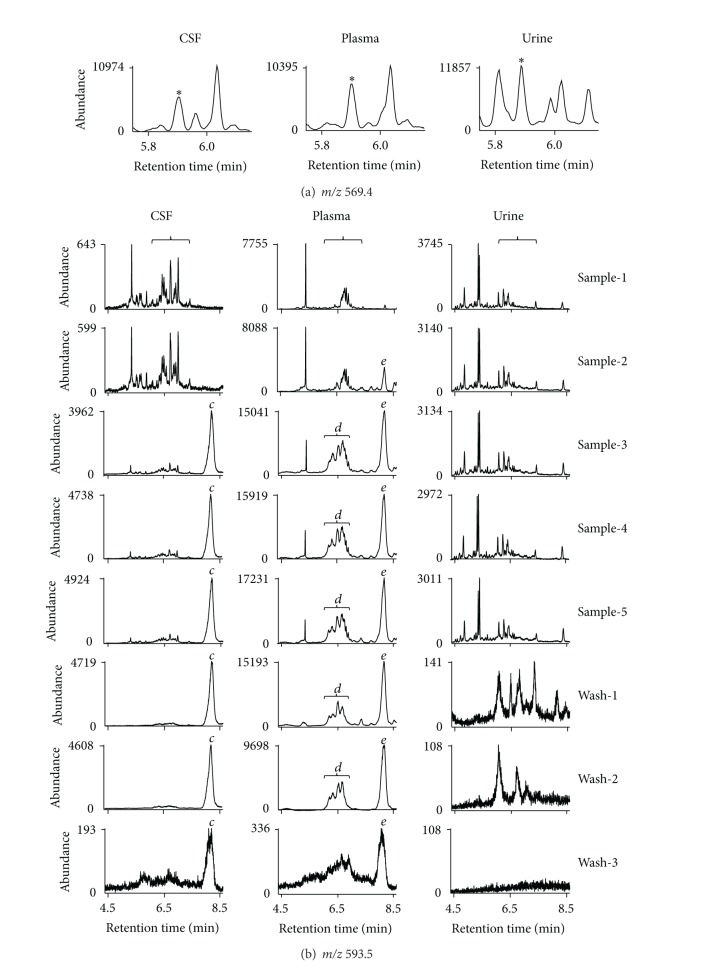
(a) Chromatograms at *m/z* 569.4 in a narrower range of retention time for the same pooled M-CSF sample, the plasma sample, and the urine sample processed for F_2_-IsoPs analysis. The peaks of F_2_-IsoPs are indicated by asterisks. The concentrations of F_2_-IsoPs quantified by the routine operation described in Section 2 were 54.9 pg/mL, 13.5 pg/mL, and 851 pg/mL for the CSF sample, the plasma sample, and the urine sample, respectively. (b) Chromatograms for the 5 consecutive injections of the same M-CSF sample (left panel), the same plasma sample (middle panel), and the same urine sample (right panel) processed for F_2_-IsoPs analysis (Sample-1 to Sample-5) and the 3 subsequent injections of undecane alone (Wash-1 to Wash-3) at *m/z* 593.5. The chromatograms in the range of 4.4 min to 8.6 min (the end of data acquisition) were compared. The range of retention time corresponding to that for F_4_-NPs quantification is indicated by the braces although these samples were not used for F_4_-NPs quantification.

 The same pattern of examination described in Section 3.2 was also applied to M-CSF, plasma, and urine samples processed for F_2_-IsoPs analysis. The chromatograms of 5 consecutive sample injections from the same pooled sample dissolved in undecane and 4 undecane washes were monitored. Although *m/z* 593.5 was not used for analysis of F_2_-IsoPs, any retained compounds detectable at *m/z* 593.5 might interfere with the analysis of F_4_-NPs of the next samples. The amount of M-CSF, plasma, and urine samples indicated in Section 2 was used and processed as for our routine analysis of F_2_-IsoPs. The chromatograms at *m/z* 569.4 in a short range of retention time showed that we had proper analysis of F_2_-IsoPs for those samples by our routine analysis (Figure 4(a)). The comparison on chromatograms in the range of 4.4 min to 8.6 min (the end of data acquisition) at *m/z* 593.5 for this test on CSF, plasma, and urine is shown by Figure 4(b), in which that for Wash-4 is not shown because of low abundance. Because the amount of the CSF sample processed for F_2_-IsoPs analysis was less than that for F_4_-NPs and the scarping area for TLC purification was also smaller, the abundance of peaks at *m/z* 593.5 was much smaller, but there was also a relatively large peak (peak *c*) at the end of chromatograms of Sample-3 to Sample-5 and Wash-1 to Wash-2 (left panel of Figure 4(b)) similar to those observed during F_4_-NPs analysis (Figure 3). Interestingly, a group of the retained peaks (*d* peaks) in the range of retention time for F_4_-NPs quantification or the large peak (peak *e*) at the end of chromatograms rises markedly for Sample-3 to Sample-5 and remained in high abundance for Wash-1 and Wash-2 (middle panel of Figure 4(b)). On the contrary, there was no apparent peak from retained substances in the chromatograms of the samples and there were only minimal signals in the chromatograms of Wash-1 and Wash-2 for urine samples (right panel of Figure 4(b)). Moreover, chromatograms of all samples and washes at *m/z* 569.4 had no obvious retention effect (see Supplemental Figure 1 of the Supplementary Material available online at http://dx.doi.org/10.1155/2013/810915), while only some peaks with low abundance showed up in Wash-1 and Wash-2 after injections of plasma samples at *m/z* 573.4 (Supplemental Figure 2). Because the amount of internal standard monitored at *m/z* 573.4 was very large, such low-abundant residual peaks should not affect the quantification of the internal standard.

### 3.4. Chromatograms Monitored during the Second Holding of the Column after Normal Acquisition of Data

**Figure 5:**
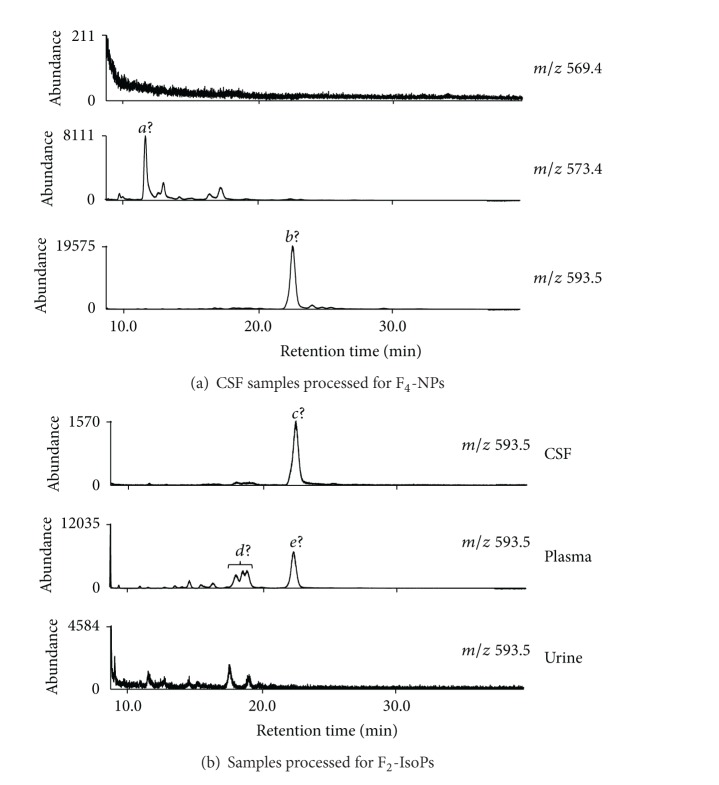
The chromatograms monitored for the additional 30-min holding (8.6 min to 38.6 min) of the column at 280°C after normal acquisition of data. (a) The chromatograms at *m/z* 569.4, *m/z* 573.4, and *m/z* 593.5 for the same M-CSF sample described in Figure 2. (b) The chromatograms at *m/z* 593.5 for the same CSF, plasma, and urine samples described in Figure 4.

 Next, we monitored possible presence of peaks in the chromatograms at all three masses representing residual substances from the first injection of samples during additional holding of the column at 280°C for 30 min after normal acquisition of data. When the same processed CSF sample used in Figure 2 was analyzed for F_4_-NPs, there was no peak with appreciable amount of abundance at *m/z* 569.4 during this second holding time (upper panel of Figure 5(a)), but significant amount of residual substances could be detected at *m/z* 573.4 and 593.5 (middle and lower panels of Figure 5(a)). Moreover, based on the patterns of the surrounding peaks, peak *a* and peak *b* indicated in Figures 2 or 3 might be present during this second holding and then moved to the retention time in the range of data acquisition during next runs. 

 The chromatograms during the second holding were also monitored for the same processed CSF, plasma, and urine samples used in Figure 4. The results showed that many peaks in substantial amount could be detected for plasma samples, whereas small but obvious peaks from the CSF and urine samples were also detectable during the additional 30-min holding at 280°C (Figure 5(b)). Peaks that were likely to be peak *c*, *d* peaks, and peak *e* were also found on the chromatograms. Moreover, there was no obvious signal above background signals at *m/z* 569.4 for CSF, plasma, and urine samples (Supplemental Figure 3(a)). The abundance of the retained peaks in the chromatograms at *m/z* 573.4 was also very low (Supplemental Figure 3(b)). 

### 3.5. Demonstration on the Elimination of Retention Effect by Additional Holding of GC Columns at a High Temperature at the End of Each Sample Analysis

 To avoid potential interferences of the retention effect on the subsequent analysis of F_4_-NPs in CSF at *m/z* 593.5, we previously needed to conduct multiple injections of undecane to monitor the conditions of GC columns, which was a troublesome process. To eliminate this problem during sample analysis in a more efficient way, we have incorporated additional holding of the column at 280°C, the highest temperature tolerated by the column recommended by the manufacturer, into the method for each injection without the detector on, which could reduce contamination of the ion source. The holding time was determined by the degree of improvement on removing retained peaks, which was confirmed by the absence of obvious peaks in the subsequent washes and disappearance of retained peaks during the second holding as what monitored in Figure 5 and in this section. 

**Figure 6:**
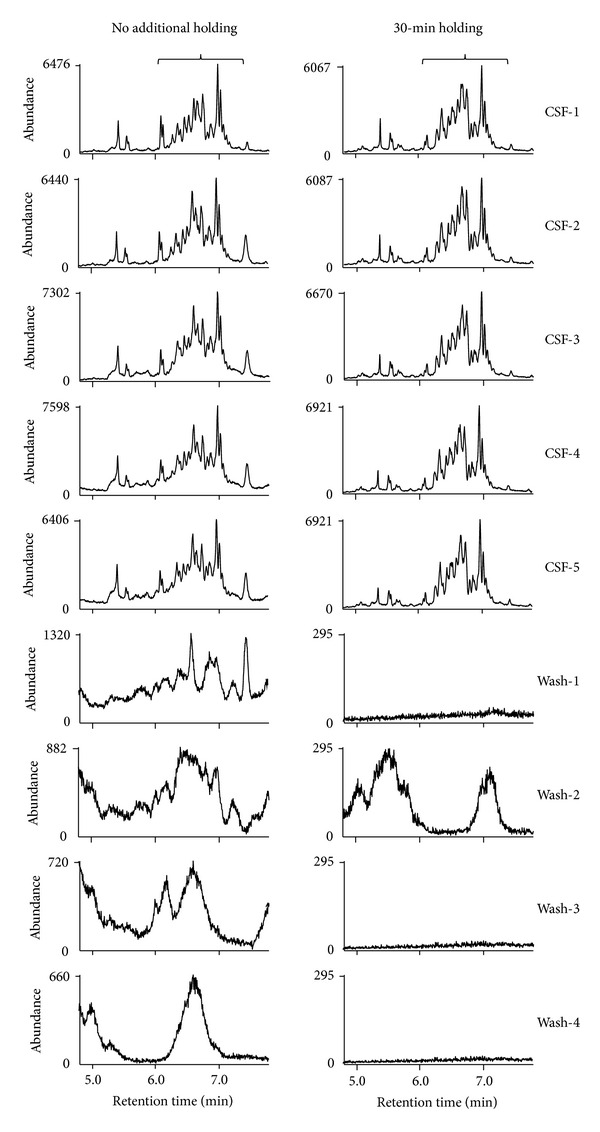
Comparison on the chromatograms for the 5 consecutive injections of the same pooled M-CSF sample processed for F_4_-NPs analysis (CSF-1 to CSF-5) at *m/z* 593.5 without (left panel) and with (right panel) additional 30-min holding of the column at 280°C at the end of data acquisition. For better viewing of the peaks of F_4_-NPs and the adjacent peaks, the chromatograms in the range of 4.8 min to 7.8 min were compared. The peaks for F_4_-NPs quantification are indicated by the braces.

 To demonstrate the effectiveness of such modification on the method setting, chromatograms for the F_4_-NPs quantification of the 5 consecutive CSF samples without and with the additional 30-min holding were compared (Figure 6). The left panel of Figure 6 is the same as the right panel of Figure 3 except showing a narrower range of retention time, which focuses on peaks for F_4_-NPs quantification. The results indicated that the patterns of F_4_-NPs peaks were gradually altered and the baseline of the peaks gradually elevated from CSF-1 through CSF-5 when there was no additional holding after acquisition of chromatograms (left panel of Figure 6). However, such interference was not found when the additional holding was conducted (right panel of Figure 6). Moreover, the large peak *b* at the end of chromatograms (right panel of Figure 3) and other small interfering peaks in the range of the retention time (left panel of Figure 6) for F_4_-NPs quantification in the chromatograms at *m/z* 593.5 of the subsequent 4 undecane washes could be observed after the CSF analysis when additional holding was not performed. That disappeared after additional 30-min holding was included in the method (right panel of Figure 6 and right panel of Supplemental Figure 4). Although there were some visible peaks in the chromatogram of Wash-2 even with additional holding, the abundance was too low to affect the quantification. On the other hand, the peak *a* and other small peaks during runs of CSF samples or washes at *m/z* 573.4 (middle panel of Figure 3) also disappeared (left panel of Supplemental Figure 4).

 The quantification results of CSF-1 to CSF-5 in Figure 6 are listed in Table 2. The trend of increase in the latter injections of samples without additional holding was clear. The quantification results were significantly greater and the within-run imprecision, presented as coefficient of variation, was higher when samples were analyzed without additional holding than that with additional holding (Table 2). In contrast, the method without the additional holding did not result in significantly higher values of F_2_-IsoPs in CSF when the same test was performed on the CSF sample processed for F_2_-IsoPs analysis (Table 3).

**Figure 7:**
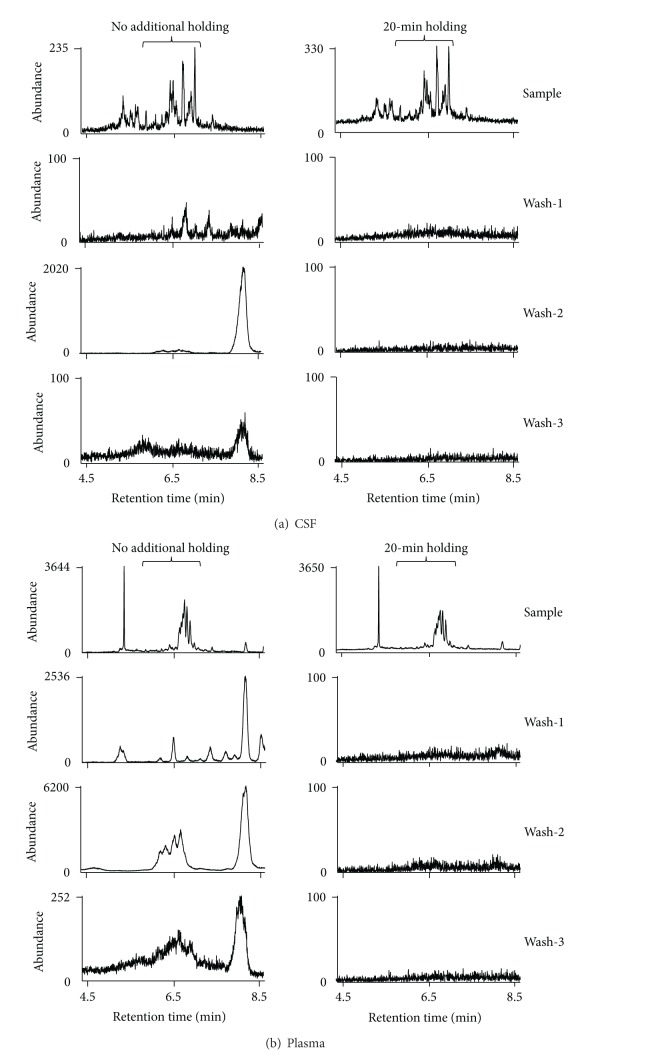
Comparison on the chromatograms at *m/z* 593.5 for one CSF sample (a) or one plasma sample (b) processed for F_2_-IsoPs analysis that has been used in Figure 4 without (left panel) and with (right panel) additional 30-min holding of the column at 280°C at the end of data acquisition. The range of retention time corresponding to that for F_4_-NPs quantification is indicated by the braces although these samples were not used for F_4_-NPs quantification.

 Furthermore, the comparison on the chromatograms in the absence and presence of second holding of the column for the same samples described for Figure 4, in which samples were processed for F_2_-IsoPs analysis, was conducted. Because chromatograms of urine samples did not exhibit any appreciable amount of peaks from retained substances that may further affect subsequences analysis of next samples at all three masses detected (Figure 4, Supplemental Figures 1, 2), this test was not conducted for urine samples. The results indicated that peaks from retained substances at *m/z* 593.5 in the chromatograms without the holding disappeared when the second holding was added in the method for both the CSF sample (Figure 7(a)) and the plasma sample (Figure 7(b)). 

## 4. Discussion

 Detection of F_2_-IsoPs has been widely applied in a large number of clinical studies, showing great utilities of this marker [16, 25]. Although quite a few of studies have detected F_2_-IsoPs in human CSF, such as our study on aSAH [17] and several publications on Alzheimer's disease (AD) [19, 21, 26], so far only three groups have measured F_4_-NPs in human CSF, which included the original report of Roberts et al. on AD [8], our studies on aSAH [13] and traumatic brain injury (TBI) [27], and the studies of Corcoran et al. on aSAH and TBI [18]. However, variations in analytical protocols and further modifications for the GC/NICI-MS analysis of these compounds without description of methods in detail or the demonstration on chromatographic data with acceptable resolution of peaks may lead to questionable results.

In this study, we demonstrated that simultaneous analysis of F_2_-IsoPs and F_4_-NPs for the human CSF samples processed for F_4_-NPs analysis caused much more overlap of the peak for F_2_-IsoPs and the adjacent peaks at *m/z* 569.4 compared with the sample that was processed for F_2_-IsoPs analysis. Consequently, the quantification results of F_2_-IsoPs became inaccurate and unreliable if CSF samples had very high levels of F_2_-IsoPs and F_4_-NPs. This indicates that proper TLC purification is critical to avoid the interferences on quantification of F_2_-IsoPs from substances with retention time that overlapped with the retention time of F_2_-IsoPs, such as other related lipid peroxidation products, PGs, and their metabolites. These compounds may not be all removed by SPE. In the literature, few studies simply quantified F_2_-IsoPs and F_4_-NPs simultaneously for brain tissues processed by the same TLC purification when using the methods of Milatovic et al. [28], Milne et al. [29], or Zhang et al. [30]. This practice may be avoided because brain tissues contain much higher amount of these compounds than CSF samples, but it might be followed for CSF samples because the availability of CSF samples is usually very limited. Great care therefore should be taken. Furthermore, although we did not perform methods described by Nourooz-Zadeh et al. [31] or Corcoran et al. [18] that analyzed F_2_-IsoPs and F_4_-NPs simultaneously for human brain tissue [31] or human CSF [18] using different methods without the step of TLC purification, the same concern raised by us should also be applied to these two platforms.

 The results from this study also revealed for the first time that significant amount of unknown compounds with low volatility were retained in the GC column from human CSF and plasma sample processed for either F_2_-IsoPs or F_4_-NPs analysis by using the regular GC elution method that originated from the methods established by the group of Roberts or Morrow [8, 11, 12, 14, 15]. Urine samples did not cause much retention effect based on the observation from the current examination possibly because we routinely started with a much less amount of urine than what was indicated in the literature. However, we cannot exclude the possibility that this phenomenon could also be enhanced by greater loading amount of samples into the GC columns even with urine samples. Although the residual substances at *m/z* 573.4 theoretically would not affect quantification of the internal standard because of the presence of relatively large amount of the internal standard, it was important to note that unknown substances at *m/z* 573.4 were also retained in the GC column substantially from CSF samples during F_4_-NPs analysis. Furthermore, many of major peaks detected during the additional holding time shown in Figure 5 appeared to show up as those peaks of the undecane washes shown in Figures 2 and 3 at the end of chromatograms or in the range of retention time for F_4_-NPs quantification. These peaks from retained substances therefore tended to move to the earlier retention time in the subsequent injections.

 The quantification of F_4_-NPs at *m/z* 593.5 for human CSF was more prone to be interfered by the retention effect from residual substances in the previous samples. When a proper method was not applied to avoid such effect, continuous injections of samples could enhance this problem by the accumulation of those substances in the GC column and enhancement of background signals. As demonstrated by our examinations, although the abundance of the interfering peaks eluted out by the undecane washes following one injection of the sample from human CSF was less than that of F_4_-NPs peaks at *m/z* 593.5 and was not likely to have a major effect on quantification of F_4_-NPs for the next sample (Figure 2), consecutive 5 injections of the same sample for F_4_-NPs quantification in the CSF sample enhanced the baseline and caused falsely higher values of F_4_-NPs (Figure 6 and Table 2), which could be prevented by the additional holding time at 280°C after normal acquisition of chromatographic data. In other words, the interfering effect from the retained substances could not be predicted by simply observing the chromatograms of undecane injections after a single sample injection. Although we did not test the potential interference on F_4_-NPs quantification in CSF following 5 consecutive analyses of plasma samples processed for F_2_-IsoPs analysis, we suspected that a group of unknown peaks (*d* peaks) in high abundance at *m/z* 593.5 from the retention of the previous plasma sample with the retention time in the range of that for F_4_-NPs peaks (Figure 4), which was much greater for those peaks from retention of the previous CSF sample processed for F_4_-NPs analysis in the same range (Figure 2), were highly likely to interfere with subsequent quantification of F_4_-NPs in CSF as well. Because this retention effect did not interfere with F_2_-IsoPs analysis at *m/z* 569.4 for CSF, plasma, or urine samples, it would not have been noticed by most researchers who only measured F_2_-IsoPs levels in human body fluids. However, this problem would be a major problem when both F_2_-IsoPs analysis and F_4_-NPs analysis were carried out interchangeably on the same GC/MS equipment without performing the additional holding at 280°C.

The time needed for the second holding of the column at the high temperature varied for different analysis and should be evaluated by observing the traces of chromatograms during the long holding after the regular acquisition of data, by comparing signals in undecane washes after sample injections with and without additional holding, and by comparing results of quantification with and without holding after multiple sample injections. The holding time therefore should also be different for different labs with different GC/MS settings or different format in sample processing, but our current study has demonstrated the rationale and necessity of such evaluation. 

 Taken together, this work has indicated the importance of proper TLC purification for obtaining reliable chromatograms for F_2_-IsoPs quantification in CSF and revealed the necessity of adding additional holding of the column at 280°C for a period of time following data acquisition during F_2_-IsoPs and F_4_-NPs analysis to avoid potential interferences on subsequent F_4_-NPs quantification in CSF. Although GC/NICI-MS is not a commonly used technique and is hard to manage, the GC/NICI-MS method remains to be the most sensitive and robust method for detecting F_2_-IsoPs or F_4_-NPs in body fluids. Many seemingly minor variations in analytical methods that may have a major impact on the reliability of results should therefore be carefully evaluated. 

## Supplementary Material

Four supplemental figures are provided to illustrate additional data described in the text. Supplemental Figure 1 and Supplemental Figure 2 show additional chromatograms at m/z 569.4 and m/z 573.4, respectively, monitored simultaneously with the data shown in Figure 4b. Supplemental Figure 3 show chromatograms at m/z 569.4 and m/z 573.4 monitored simultaneously with the data shown in Figure 5b using the same samples described in Figure 4. Supplemental Figure 4 show chromatograms at 573.4 monitored simultaneously with the data shown in right panel of Figure 6 (m/z 593.5), which employed the same samples described in Figure 3, along with chromatograms at 593.5 having broader range of retention time than what shown in Figure 6.

## Figures and Tables

**Table 1:** Results of quantification of F_2_-IsoPs and F_4_-NPs in three test CSF samples that have been processed for F_2_-IsoPs (IsoP method) or F_4_-NPs (NP method) analysis.

	NP method		IsoP method	% of difference for F_2_-IsoPs^c^
	F_4_-NPs (pg/mL)	F_2_-IsoPs (pg/mL)	F_2_-IsoPs (pg/mL)	
L-CSF	57.7 ± 5.1 (8.8%)	19.5 ± 0.1 (0.5%)	20.6 ± 0.4 (1.7%)^a^	5.2%
M-CSF	109.0 ± 9.9 (9.1%)	56.9 ± 1.7 (3.0%)	55.9 ± 0.9 (1.7%)	1.7%
H-CSF	1023.4 ± 116.7 (11.4%)	141.0 ± 4.0 (2.8%)	85.0 ± 0.8 (0.9%)^b^	66.0%

There were three replicates for each CSF sample. Nine CSF samples were processed and analyzed together. Data were presented as mean ± standard deviation (coefficient of variation). Coefficient of variation was the ratio of standard deviation to mean presented as percentage and was used to represent within-run imprecision. ^a^ *p* < 0.05 (*p* = 0.007 under assumption of equal variance versus values of F_2_-IsoPs for L-CSF processed by the NP method). ^b^ *p* < 0.05 (*p* = 0.001 under assumption of unequal variance versus values of F_2_-IsoPs for H-CSF processed by the NP method). ^c^The difference between the mean values of two methods divided by the mean values from IsoP method is calculated and represented as % of difference.

**Table 2:** Results of quantification of F_4_-NPs from 5 consecutive injections of the same pooled sample.

	F_4_-NPs levels (pg/mL)	
	No additional holding	30-min holding
CSF-1	119.9	113.8
CSF-2	130.2	114.0
CSF-3	144.4	117.7
CSF-4	145.5	119.3
CSF-5	147.5	121.7

Mean ± SD	137.5 ± 12.0	117.3 ± 3.4*
(CV%)	(8.7%)	(2.9%)

The sample number was designated according to the sequence of injection. The quantification data for samples analyzed without additional holding corresponds to chromatograms shown by the left panel of Figure 6, whereas that with 30-min holding corresponds to chromatograms shown by the right panel of Figure 6. CV is coefficient of variation that is the ratio of standard deviation (SD) to mean. *p < 0.05 (*p* = 0.017 under assumption of equal variance) between two sets of data obtained by two different methods.

**Table 3:** Results of quantification of F_2_-IsoPs from 5 consecutive injections of the same pooled sample.

	F_2_-IsoPs levels (pg/mL)	
	No additional holding	30-min holding
CSF-1	56.1	54.8
CSF-2	55.5	55.3
CSF-3	54.8	55.2
CSF-4	54.1	56.4
CSF-5	53.8	55.5

Mean ± SD	54.9 ± 1.0	55.4 ± 0.6
(CV%)	(1.8%)	(1.1%)

The sample number was designated according to the sequence of injection. There was no statistical significance between two sets of data obtained by two different methods (*p* = 0.281 under assumption of equal variance).
