# Supplementary material for: Analytical Variables Affecting Analysis of F2-Isoprostanes and F4-Neuroprostanes in Human Cerebrospinal Fluid by Gas Chromatography/Mass Spectrometry
Source: Biomed Res Int. 2013 Jul 10;2013:810915. doi: 10.1155/2013/810915 (PMC3727202; doi:10.1155/2013/810915)
Supplement: Supplementary file 1 — Four supplemental figures are provided to illustrate additional data described in the text. Supplemental Figure 1 and Supplemental Figure 2 show additional chromatograms at m/z 569.4 and m/z 573.4, respectively, monitored simultaneously with the data shown in Figure 4b. Supplemental Figure 3 show chromatograms at m/z 569.4 and m/z 573.4 monitored simultaneously with the data shown in Figure 5b using the same samples described in Figure 4. Supplemental Figure 4 show chromatograms at 573.4 monitored simultaneously with the data shown in right panel of Figure 6 (m/z 593.5), which employed the same samples described in Figure 3, along with chromatograms at 593.5 having broader range of retention time than what shown in Figure 6. [file 810915.f1.zip › 810915.f1/supplementery figure 1.pdf]

Supplemental Figures

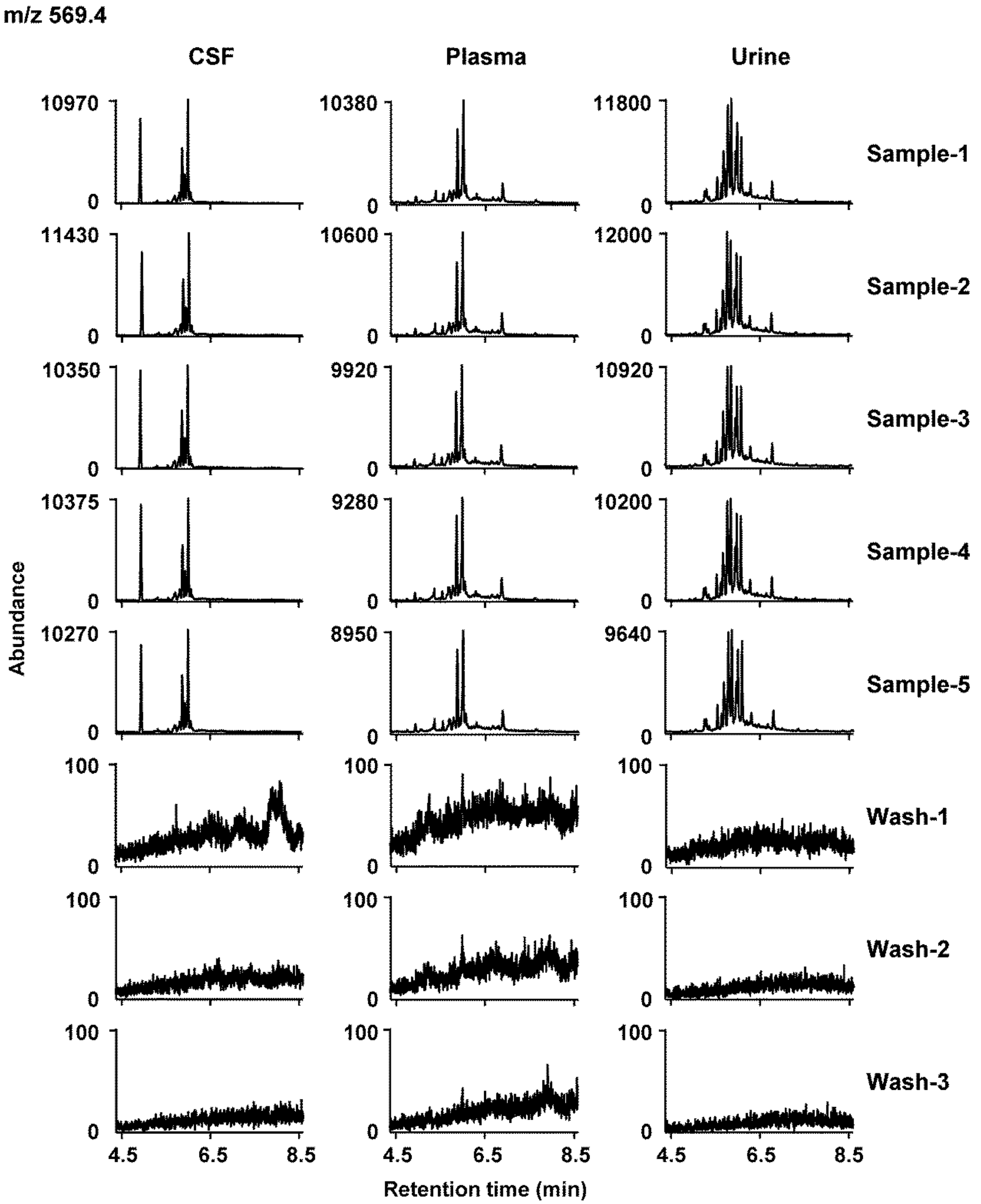

Supplemental Figure 1. Chromatograms for the 5 consecutive injections of the same M-CSF sample (left panel), the same plasma sample (middle panel), and the same urine sample (right panel) processed for F_2-IsoPs analysis (Sample-1 to Sample-5) and the 3 subsequent injections of undecane alone (Wash-1 to Wash-3) at m/z 569.4. They were monitored simultaneously with data shown in Figure 4B described in Figure 3. The chromatograms in the range of 4.4 min to 8.6 min (the end of data acquisition) were compared.
