# Supplementary material for: Analytical Variables Affecting Analysis of F2-Isoprostanes and F4-Neuroprostanes in Human Cerebrospinal Fluid by Gas Chromatography/Mass Spectrometry
Source: Biomed Res Int. 2013 Jul 10;2013:810915. doi: 10.1155/2013/810915 (PMC3727202; doi:10.1155/2013/810915)
Supplement: Supplementary file 1 — Four supplemental figures are provided to illustrate additional data described in the text. Supplemental Figure 1 and Supplemental Figure 2 show additional chromatograms at m/z 569.4 and m/z 573.4, respectively, monitored simultaneously with the data shown in Figure 4b. Supplemental Figure 3 show chromatograms at m/z 569.4 and m/z 573.4 monitored simultaneously with the data shown in Figure 5b using the same samples described in Figure 4. Supplemental Figure 4 show chromatograms at 573.4 monitored simultaneously with the data shown in right panel of Figure 6 (m/z 593.5), which employed the same samples described in Figure 3, along with chromatograms at 593.5 having broader range of retention time than what shown in Figure 6. [file 810915.f1.zip › 810915.f1/supplementery figure 3.pdf]

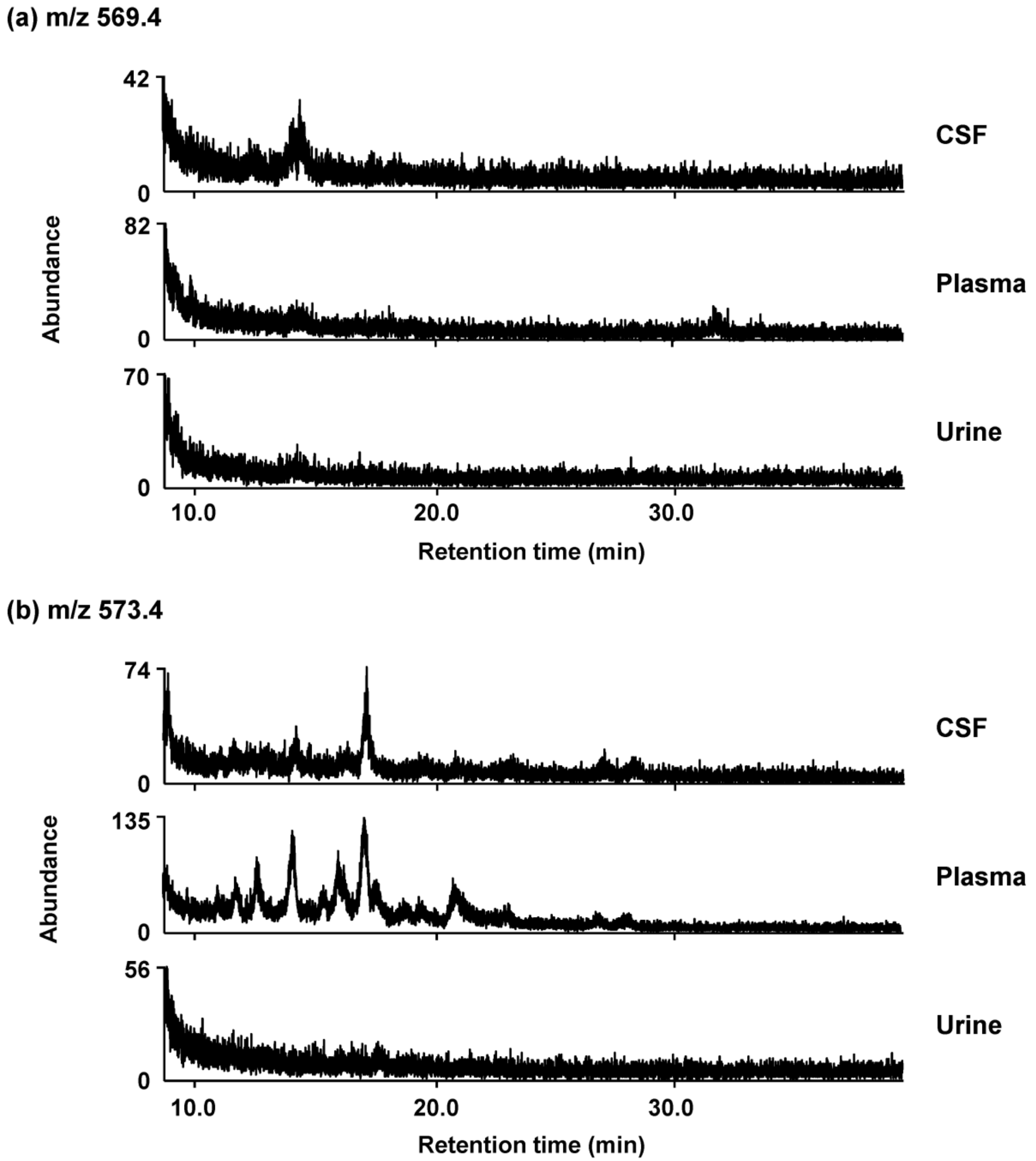

Supplemental Figure 3. The chromatograms monitored for the additional 30-min holding (8.6 min to 38.6 min) of the column at 280℃ after normal acquisition of data. (a) The chromatograms at m/z 569.4 for the same CSF, plasma, and urine samples described in Figure 4. (b) The chromatograms at m/z 573.4 for the same CSF, plasma, and urine samples described in Figure 4.
